# Ion homeostasis in a salt-secreting halophytic grass

**DOI:** 10.1093/aobpla/plv055

**Published:** 2015-05-19

**Authors:** Payal Sanadhya, Parinita Agarwal, Pradeep K. Agarwal

**Affiliations:** 1 Wasteland Research Division, CSIR-Central Salt and Marine Chemicals Research Institute (CSIR-CSMCRI), Council of Scientific and Industrial Research (CSIR), Gijubhai Badheka Marg, Bhavnagar 364 002, Gujarat, India; 2 Academy of Scientific and Innovative Research, CSIR-Central Salt and Marine Chemicals Research Institute (CSIR-CSMCRI), Council of Scientific and Industrial Research (CSIR), Gijubhai Badheka Marg, Bhavnagar 364 002, Gujarat, India

**Keywords:** *Aeluropus*, ion homeostasis, ion transporters, osmotic adjustment, salt secretion, transcript profiling

## Abstract

Salinity adversely affects plant growth and development, and disturbs intracellular ion homeostasis, resulting in cellular toxicity. Plants that tolerate salinity, halophytes, do so by manifesting numerous physiological and biochemical processes in coordination to alleviate cellular ionic imbalance. The present study was undertaken to analyse the salt tolerance mechanism in *Aeluropus lagopoides* (L.) trin. Ex Thw. (Poaceae) at both physiological and molecular levels. Plants secreted salt from glands, which eventually produced pristine salt crystals on leaves and leaf sheaths. The rate of salt secretion increased with increasing salt concentration in the growth medium. Osmotic adjustment was mainly achieved by inorganic osmolytes (Na^+^) and at 100 mM NaCl no change was observed in organic osmolytes in comparison to control plants. At 300 mM NaCl and with 150 mM NaCl + 150 mM KCl, the concentration of proline, soluble sugars and amino acids was significantly increased. Transcript profiling of transporter genes revealed differential spatial and temporal expressions in both shoot and root tissues in a manner synchronized towards maintaining ion homeostasis. In shoots, *AlHKT2;1* transcript up-regulation was observed at 12 and 24 h in all the treatments, whereas in roots, maximum induction was observed at 48 h with K^+^ starvation. The *HAK* transcript was relatively abundant in shoot tissue with all the treatments. The plasma membrane Na^+^/H^+^ antiporter, *SOS1*, and tonoplast Na^+^/H^+^ antiporter, *NHX1*, were found to be significantly up-regulated in shoot tissue. Our data demonstrate that *AlHKT2;1*, *HAK*, *SOS1*, *NHX1* and *V-ATPase* genes play a pivotal role in regulating the ion homeostasis in *A. lagopoides*.

## Abstract (precis)

Salinity adversely affects plant growth and development, and disturbs intracellular ion homeostasis, resulting in cellular toxicity. Glycophytes tolerate salinity up to 40 mM NaCl, however halophytes grow luxuriantly in salt-marshes where the salt concentration is 200 mM NaCl or more. *Aeluropus lagopoides* secretes salt crystals from the adaxial and abaxial leaf surface through salt glands. At high salt concentrations the level of different organic and inorganic osmolytes is elevated to prevent toxicity and maintain a sustainable K^+^/Na^+^ ratio. Different transporter genes *AlHKT2;1*, *HAK, SOS1, NHX1,* and *V-ATPase* expression coordinate to regulate ion homeostasis in *A. lagopoides*.

## Introduction

Plants being sessile are negatively affected in unfavourable environmental conditions. Salinity, one of the major stresses that cause substantial damage to crop productivity, leads to osmotic stress at an early phase and ionic stress at a later phase of plant growth (Munns and Tester 2008), activating an array of changes at morphological, physiological, biochemical and molecular levels. Plants employ different mechanisms to overcome salinity stress, such as ion compartmentalization, osmotic adjustment, selective ion uptake and transport, succulence and salt inclusion/secretion (Flowers and Colmer 2008). The maintenance of ion homeostasis is a prerequisite for survival in highly saline environments. Transmembrane ionic movement is a well-balanced event with net influx adjusted to accommodate cellular requirements, and maintaining ion homeostasis via specialized transporter proteins that are generally categorized as pumps, carriers and channels (Amtmann and Sanders 1999).

Ion homeostasis is maintained by membrane transporters like SOS1 (salt overly sensitive), NHX1 (Na^+^/H^+^ exchanger), H^+^-ATPase (Shi *et al*. 2002; Su *et al*. 2007), HAK (high-affinity K^+^ transporter; Maathuis 2006) and HKT (high-affinity K^+^ transporter; Horie *et al*. 2009). The plasma membrane Na^+^/H^+^ antiporter, SOS1, mediates the extrusion of Na^+^ from cytoplasm to the apoplastic region (Hasegawa 2013) and is also involved in long-distance Na^+^ transport from root to shoot (Shi *et al*. 2002). NHX1-type antiporters are localized in the tonoplast and facilitate the removal of Na^+^ from the cytosol and its subsequent sequestration into the vacuoles (Hasegawa 2013). Both the SOS1 and NHX1 antiporters are energized by the proton motive force (PMF) generated by the H^+^-ATPase proton pump and the PPiase (Hasegawa 2013). Several HAK (Maathuis 2006) and HKT (Hauser and Horie 2010) transporters mediate high-affinity K^+^ uptake during salt stress against an electrochemical gradient and consequently help the plants to maintain their K^+^/Na^+^ balance.

Halophytes have developed adaptability to survive and complete their life cycle in saline environments, and therefore understanding the mechanism of their tolerance is at the forefront of research in salinity tolerance. The halophyte *Aeluropus lagopoides* (L.) trin. Ex Thw. belongs to the Poaceae, a family with many species important as food grains. *Aeluropus lagopoides* is mainly distributed in Northern Africa (Morocco to Somalia), Sicily, Cyprus, Central Asia, Pakistan and India (Gulzar and Khan 2001). It is a C4 salt-secreting perennial grass, growing luxuriously on the muddy banks of creeks and in the adjacent intertidal areas of Gujarat, India. It survives at even 1 M NaCl, although, growth is greatly reduced at >300 mM NaCl (Gulzar *et al*. 2003). Different adaptations like fast growth rate, rapid propagation of stems by means of rhizomes, deep and widespread root network, abundant seed production, small leaves and salt secretion help this grass to survive under high salt concentration (Mohsenzadeh *et al*. 2006). This plant serves as a cattle feed because of the low sodium content in the shoots (Torbatinejad *et al*. 2000). Previous ecological and physiological studies on *A. lagopoides* suggested that salt stress leads to reduced plant growth, photosynthesis, K^+^ content and soluble protein and increased accumulation of proline and Na^+^ (Sobhanian *et al*. 2010*a*, *b*; Ahmed *et al*. 2013). Sobhanian *et al*. (2010*a*) through a proteomic approach revealed that during salt stress in *A. lagopoides*, metabolism-related proteins involved in energy production and amino acid biosynthesis were up-regulated, and photosynthesis-related proteins were down-regulated.

Most of the previous studies have focussed on physiological and biochemical responses of *A. lagopoides* during salt stress. However, meagre information is available on the regulation of different ion transporters during salt stress and K^+^ starvation. Therefore, the present work was carried out to study the role of Na^+^ and K^+^ transporter genes during different salt stresses (0, 100 mM NaCl, 300 mM NaCl, 150 mM NaCl + 150 mM KCl) and K^+^ starvation. It was observed that *A. lagopoides* secretes salts from its salt glands and the rate of secretion increases concomitantly with increasing salt concentration to maintain balanced Na^+^ concentration. Furthermore, gene expression profiling revealed that different ion transporters work in a coordinated manner to maintain ion homeostasis in this plant.

## Methods

### Plant growth and salt treatments

**Figure 1.**
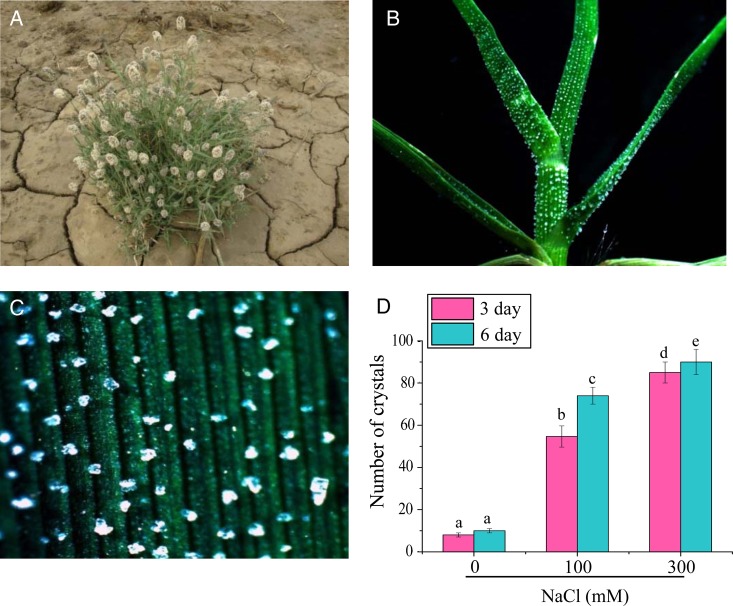
(A) *Aeluropus lagopoides* plants growing in natural habitat. (B) Photograph showing secretion of ions from leaf sheath and leaf surfaces. (C) Salt crystals on the adaxial leaf surface. (D) Crystal count on leaves of *A. lagopoides* in NaCl stress. The mean values significantly different at *P* ≤ 0.05 within and among treatments are indicated by different letters. The error bars indicate the SD.

*Aeluropus lagopoides* plants (Fig. 1A) were collected from the CSIR-CSMCRI salt farm, Bhavnagar, Gujarat (N21°47′13.5″; E72°07′25.7″), India. Nodal cuttings with 2–3 pairs of leaves were excised from the runners and planted in half-strength hydroponics Hoagland's medium (Hoagland and Arnon 1950) in plastic pots (diameter × height: 21 × 7 cm) and kept in a growth chamber with a dark/light (300–350 µmol m^−2^ s^−1^ of photosynthetically active radiations) cycle of 16/8 h at 25 °C. After 7 days, morphologically similar plantlets were exposed to ionic stress by directly subjecting them to the following treatments: (i) 100 mM NaCl, (ii) 300 mM NaCl, (iii) 150 mM NaCl + 150 mM KCl and (iv) K^+^ starvation. For K^+^ starvation, Hoagland medium was modified by removing the potassium nitrate, while the nitrate source was maintained with an additional amount (0.64 g L^−1^) of Ca(NO_3_)_2_. Three independent biological replicates were used for all the experiments. For transcript profiling, plant samples were harvested at 12, 24 and 48 h after initiation of stress, as the transcript of transporter genes is reported to show early induction (Kader *et al*. 2006) when compared with biochemical and physiological responses. The samples were rapidly frozen in liquid N_2_ and stored at −80 °C until used for RNA isolation. For biochemical analysis, shoots were harvested after 3 and 6 days for each treatment, frozen in liquid nitrogen and stored at −80 °C.

### Scanning electron micrography (SEM) of the crystals

Second leaves from the top of the field-grown plants were scanned and photographed with a scanning electron microscope (FEI quanta 200) equipped with an energy-dispersive X-ray (EDX) system.

### Crystal count

Plantlets of *A. lagopoides* were subjected to 0, 100 and 300 mM NaCl for 3 and 6 days. Photographs were taken from the middle portion of three leaves from each treatment at the same magnification using a stereomicroscope (Leica L2) and crystals counted. The experiment was repeated three times and significantly different mean values at *P* ≤ 0.05 within and among treatments are indicated by different letters.

### Tissue water content

The plantlets were harvested after 6 days and separated into shoots and roots. The samples were dried by wrapping in tissue paper and the fresh weight (FW) was measured just after harvesting the plants; dry weight (DW) was measured after drying the samples at 70 °C until a constant weight was attained. Tissue water content (TWC) was calculated on dry weight basis: (FW − DW)/DW.

### Ion content analysis

For ion content determination, the shoot and root samples were harvested after 6 days for each treatment, rinsed with de-ionized water and dried at 70 °C. Dried samples were digested with 4 mL of perchloric acid and nitric acid solution (3 : 1) (Shukla *et al*. 2012). The contents of Na^+^, K^+^ and Ca^2+^ in the shoots and roots was determined by inductively coupled plasma optical emission spectrometer (Optima 2000DV, PerkinElmer, Germany).

### Compatible solutes

Total soluble sugars (TSS), total amino acids (TAA) and proline were determined in the shoots from 95 % ethanolic extracts. Frozen tissue was crushed in 5 mL of 95 % ethanol. The extract was vortexed and centrifuged at 3500 rpm for 10 min. The pellet was washed twice with 5 mL of 70 % ethanol. All soluble portions were pooled and centrifuged to remove debris and the supernatant was stored at 4 °C (1 week) for further analysis.

Total soluble sugars were estimated colourimetrically at 630 nm with freshly prepared anthrone reagent (Irigoyen *et al*. 1992). An aliquot of 100 μL ethanolic extract was added to 3 mL of anthrone–sulfuric acid reagent (200 mg anthrone dissolved in 100 mL 72 % H_2_SO_4_) and incubated in a boiling water bath for 10 min and cooled to room temperature. The absorbance was measured at 630 nm (Epoch Micro-Volume spectrophotometer, Biotek, India). A standard graph was plotted using various concentrations of glucose in the range of 20–200 μg mL^−1^.

Total amino acids were analysed according to Shukla *et al*. (2012). The reaction mixture comprised 1 mL each of the ethanolic extract, 0.2 M citrate buffer (pH 5), 80 % ethanol and 1 % ninhydrin. The mixture was vortexed and incubated in a boiling water bath for 15 min. The absorbance was measured at 570 nm and amino acid concentration was expressed as μmol mg^−1^ FW.

Free proline was quantified in the shoot tissues using ninhydrin as described by Carillo *et al*. (2008). Ethanolic extract (100 μL) was mixed with 200 μL of reaction mix [1 % ninhydrin (w/v) in 60 % acetic acid (v/v) and 20 % ethanol (v/v)] and incubated in a water bath at 95 °C. Absorbance of the reaction mixture was determined at 520 nm and the free proline concentration estimated using a standard graph drawn from known concentrations of l-proline.

### Oxidative stress markers

Lipid peroxidation was measured by estimation of total 2-thiobarbituric acid reactive substances (TBARS) and expressed as malondialdehyde (MDA) equivalents (Hodges *et al*. 1999). Shoot samples were ground in 15 mL of 80 % ethanol and centrifuged at 3500 rpm for 10 min. In one set, 1 mL of extract was added to 1 mL 0.5 % thiobarbituric acid (TBA) in 20 % trichloroacetic acid (TCA). In another set, TBA was excluded. The mixture was incubated at 90 °C for 30 min and subsequently cooled at room temperature. The samples were centrifuged at 4000 rpm for 5 min and the absorbance of the supernatants was read at 440, 532 and 600 nm. Malondialdehyde equivalents (nmol mL^−1^) were determined by the formula given by Hodges *et al*. (1999).

$$[(\text{Abs}\;532_{+\mathrm{TBA}})-(\text{Abs}\;600_{+\mathrm{TBA}})-(\text{Abs}\;532_{-\mathrm{TBA}}-\text{Abs}\;600_{-\mathrm{TBA}})]=A$$

$$[(\text{Abs}\;440_{+\mathrm{TBA}}-\text{Abs}\;600_{+\mathrm{TBA}})\;0.0571]=B$$

$$\text{MDA}\;\text{equivalents}\;(\text{nmol}\;\text{m}{\text{L}}^{-1})=(A-B/157\;000)\times 10^{6}$$

The rate of generation of ${{\text{O}}_{2}}^{-}$ radical was determined according to Elstner and Heupel (1976). Shoot tissue was homogenized in 10 mL of 65 mM potassium phosphate buffer (pH 7.8) and centrifuged at 5000 rpm for 10 min. The mixture containing 1 mL of extract, 0.9 mL of 65 mM phosphate buffer (pH 7.8) and 0.1 mL of 10 mM hydroxyl amine hydrochloride was incubated at 25 °C for 20 min. The colour was developed by addition of 17 mM sulfanilamide and 7 mM α-naphthylamine and further incubation at 25 °C for 20 min. The specific absorbance of the samples and standards was recorded at 530 nm. Sodium nitrite was used as a standard (10–200 nmol) for plotting the standard graph.

The hydrogen peroxide (H_2_O_2_) concentration in the shoots was determined as described by Mukherjee and Choudhuri (1983). Shoot tissue was homogenized in cold acetone and 1 mL of the extract mixed with 0.5 mL of titanium reagent (0.1 % titanium dioxide dissolved in 20 % H_2_SO_4_) followed by centrifugation at 6000 rpm for 15 min. The intensity of yellow colour in the reaction mixture was measured at 415 nm and the concentration of H_2_O_2_ calculated against the standard curve of H_2_O_2_.

### Real-time PCR

Total RNA was isolated from shoot and root tissues of control and various stress-treated *A. lagopoides* plantlets by the GITC extraction protocol (Chomczynski and Sacchi 1987). RNA (2 μg) was treated with DNaseI (MBI Fermentas) followed by first-strand cDNA synthesis with a RevertAid first-strand cDNA synthesis kit (Thermo Scientific). The transcript expression of *AlHKT2;1*, *HAK*, *SOS1*, *NHX1* and *V-ATPase* genes was studied by real-time PCR using gene-specific primers designed from the sequences available at NCBI (Table 1). Real-time PCR was performed by 1× Sso Advanced SYBR green supermix (Bio-Rad, USA) on the CFX96 real-time system (Bio-Rad) as per the manufacturers’ instructions. The reverse transcriptase PCR was done using cDNA as template with the following PCR conditions: 94 °C, 4 min for 1 cycle; 94 °C, 1 min; 55 °C, 1 min and 72 °C, 1 min for 30 cycles and final extension at 72 °C, 7 min. The relative fold change in gene transcript was calculated by the comparative *C*_t_ $\left(2^{-\Delta \Delta C_{t}}\right)$ method using actin as an internal reference gene (Livak and Schmittgen 2001).

**Table 1.** Primer sequences for the genes selected for real-time PCR.

Gene	Primer name	Sequence (5′–3′)	Accession number
Actin	AlActinF	5′-TACGAAGGGTTTACGCTTCCT-3′	GW796822
	AlActinR	5′-TCTCCAACTCCTCCTCGTAAT-3′	
AlHKT2;1	AlHKTF	5′-GTTCAGTCCTCTTGATGTCGCT-3′	KP081769
	AlHKTR	5′-GCGCTCTTGCAGATGGTACTTGG-3′	
HAK	AlHAKF	5′-ACTGTCAAAGTTCATCGAGGG-3′	DQ645465
	AlHAKR	5′-GGCAGGTGCTTGATCGACATGAAC-3′	
SOS1	AlSOS1F	5′-CCTTCAGATGAGTAGGCTGCCACG-3′	JN936862
	AlSOS1R	5′-GTCAATACCGAGTATGTTACTTG-3′	
NHX1	AlNHXF	5′-AAGATCGATGTAGCCGTTGTAC-3′	GU199336
	AlNHXR	5′-TCTGATAGCTCAGCCAGCATGT-3′	
V-ATPase	AlATPaseF	5′-ACTGATCTTGGAGGACTGCAAG-3′	GW796823
	AlATPaseR	5′-TCGACAGCAGGATAAATACCAAG-3′	

### Statistical analysis

Each experiment was repeated three times; the mean values and standard deviations were calculated. Two-way analysis of variance (ANOVA) (Figs 1D, 3 and 5–7; Tables 2 and 3) and one-way ANOVA (Fig. 4; Table 4) were performed using Microsoft Excel and critical difference values were calculated at *P* ≤ 0.05 to determine the significance of difference between the means of control and different stress treatments. Mean values that were significantly different from each other and among the treatments were indicated by different letters in the graphs.

**Table 2.** Analysis of variance of (two-way) different parameters to represent the variation within and between treatments at *P* ≤ 0.05.

Parameters	Between treatments		Within treatments		*F* value	*F*_crit_
	DF	MS	DF	MS		
Crystal counting	2	139.5	12	16.38	8.51	3.89
Dry weight	4	19.97	20	7.80	2.5	2.87
TWC	4	1.217	20	0.108	11.26	2.87
TSS	4	1044.91	20	79.13	13.20	2.87
TAA	4	0.092	20	0.011	7.87	2.87
Proline	4	45.06	20	0.277	162.56	2.87
Superoxide	4	0.015724	20	0.002	6.42	2.87
Peroxide	4	158.04	20	74.32	2.12	2.87
MDA	4	373.01	20	3.14	118.78	2.87

**Table 3.** Analysis of variance of (two-way) transcript regulation of different genes to represent the variation within and between treatments at *P* ≤ 0.05.

Genes	Between treatments		Within treatments		*F* value	*F*_crit_
	DF	MS	DF	MS		
Shoot						
AlHKT2;1	6	18.66	24	1.22	15.28	2.51
HAK	6	1.20	24	0.09	12.79	2.51
SOS1	6	373.71	24	2.33	160.05	2.51
NHX1	6	50.36	24	1.02	49.36	2.51
V-ATPase	6	0.41	24	0.01	33.02	2.51
Root						
AlHKT2;1	6	16.48	24	0.08	198.60	2.51
HAK	6	1.58	24	0.04	36.84	2.51
SOS1	6	0.47	24	0.01	38.83	2.51
NHX1	6	4.23	24	0.05	80.31	2.51
V-ATPase	6	6.29	24	0.04	159.77	2.51

**Table 4.** Analysis of variance of (one-way) ion analysis to represent the variation within and between treatments at *P* ≤ 0.05.

	Between groups		Within groups		*F* value	*F*_crit_
	DF	MS	DF	MS		
Sodium shoot	4	0.193	10	0.0050	38.92	3.48
Sodium root	4	0.526	10	0.0063	84.08	3.48
Potassium shoot	4	0.220	10	0.0042	51.92	3.48
Potassium root	4	0.408	10	0.0051	80.17	3.48
Calcium shoot	4	0.001	10	0.0007	1.01	3.48
Calcium root	4	0.009	10	0.0005	17.35	3.48

## Results

### Exudation of salt crystals

**Figure 2.**
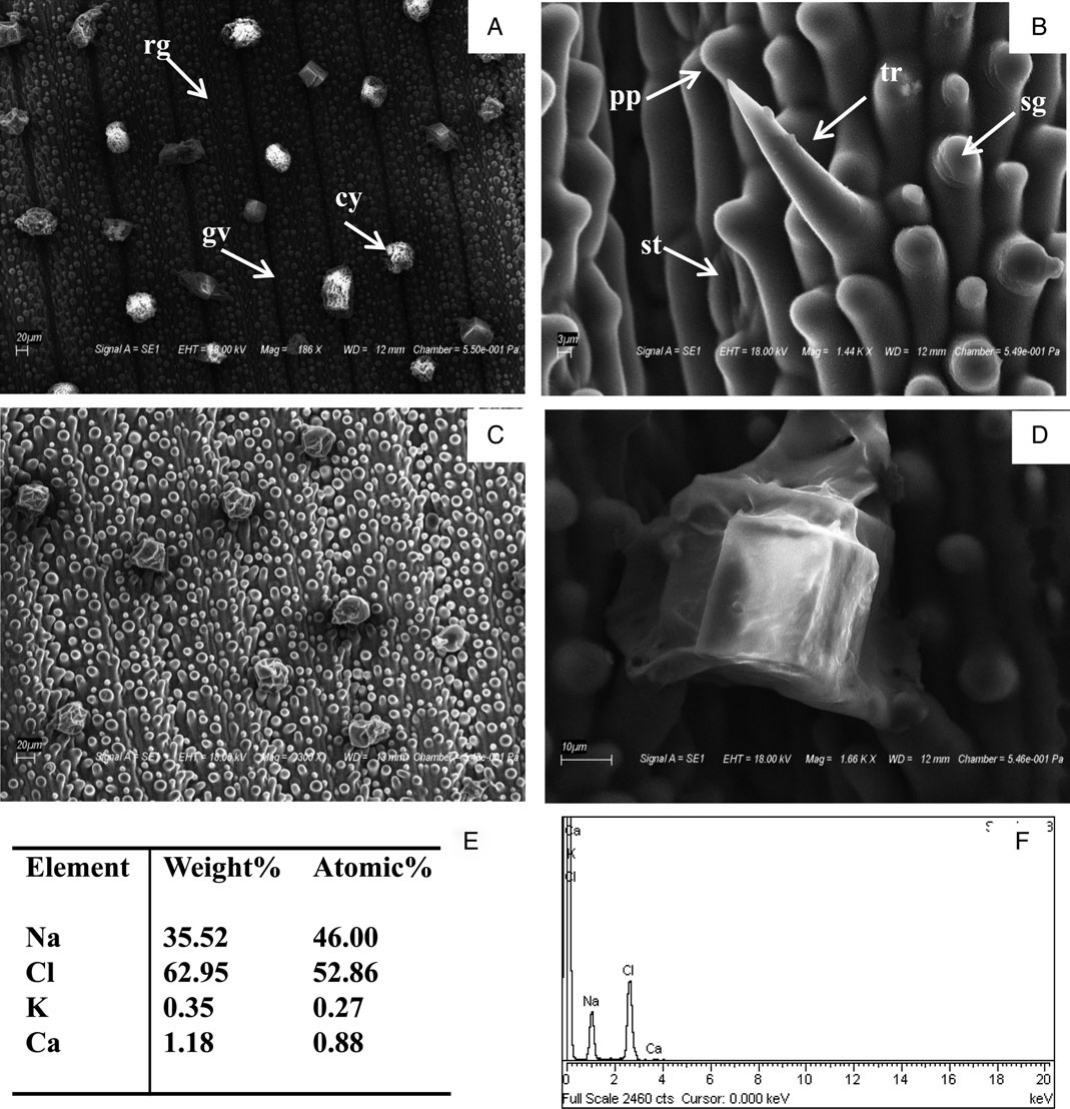
The SEM image of leaves showing different epidermal structures: (A) grooves (gv), ridges (rg) and crystals (cy) on the adaxial leaf surface; (B) adaxial leaf surface showing trichomes (tr) papillae (pp), salt glands (sg) and stomata (st); (C) a view of the abaxial leaf surface. Gv, rg and tr were found on the adaxial side; however, they were absent on the abaxial surface. Pp, sg and st were seen uniformly on both sides of the leaf surfaces. (D) Enlarged view of a asingle salt crystal from the adaxial leaf surface, (E) element analysis and (F) typical spectrum of the SEM X-ray microanalysis from the crystal.

For analysis of salt crystals, plants were grown in Hoagland's medium in hydroponic culture conditions. These plants showed salt crystals on the leaf sheath as well as adaxial and abaxial leaf surfaces (Fig. 1B). The number of salt crystals was systematically examined after 3 and 6 days at 0, 100 and 300 mM NaCl. The salt crystals appeared at the leaf ridges in horizontal rows (Fig. 1C). At 0 mM NaCl, very few crystals were observed but with increasing NaCl concentration the number of crystals increased (Fig. 1D). A significant difference in the number of crystals was observed after 3 and 6 days in 100 and 300 mM NaCl. The adaxial and abaxial surfaces showed notable morphological difference in the SEM micrographs. The adaxial surface was undulated and showed alternatively arranged longitudinal ridges and furrows (Fig. 2A). Short epidermal structures (trichomes) with pointed ends (Fig. 2B) were present on the adaxial surfaces along the ridges. The abaxial surface was marked by the absence of ridges (Fig. 2C). Papillae, salt glands and stomata were observed on both the surfaces. Trichomes were not observed on the abaxial surface (Fig. 2C). The salt crystals observed under SEM showed a cubic structure (Fig. 2D) and revealed the composition of Na^+^ and Cl^−^ by SEM EDX (Fig. 2E and F).

### Dry weight and TWC

**Figure 3.**
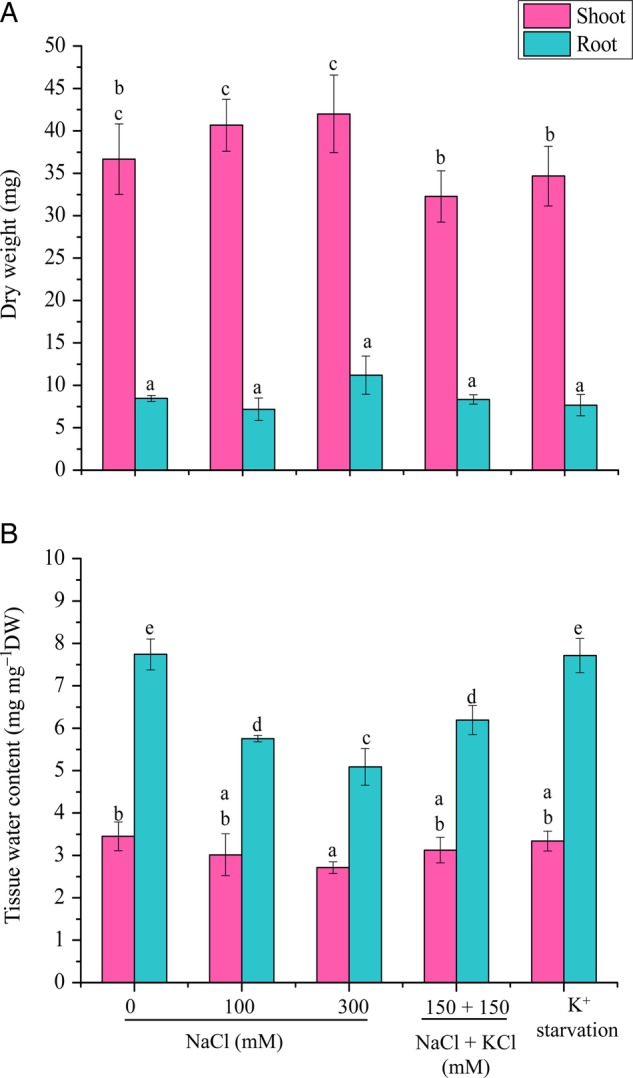
Effect of different stress treatments on (A) DW and (B) TWC after 6 days. The mean values significantly different at *P* ≤ 0.05 within and among treatments are indicated by different letters. The error bars indicate the SD.

The shoot DW was significantly increased in 100 and 300 mM NaCl treatments compared with the control (Fig. 3A). No significant changes were observed in the root DW in response to different stress treatments when compared with the control (Fig. 3A). Tissue water content was reduced significantly in both shoot and root tissue from 0 to 300 mM NaCl. However, no significant change in TWC was observed in shoots at 100 mM NaCl, 150 mM NaCl + 150 mM KCl and K^+^ starvation. In root tissue all the treatments showed significant difference except K^+^ starvation in comparison to control (Fig. 3B).

### Ion content analysis

**Figure 4.**
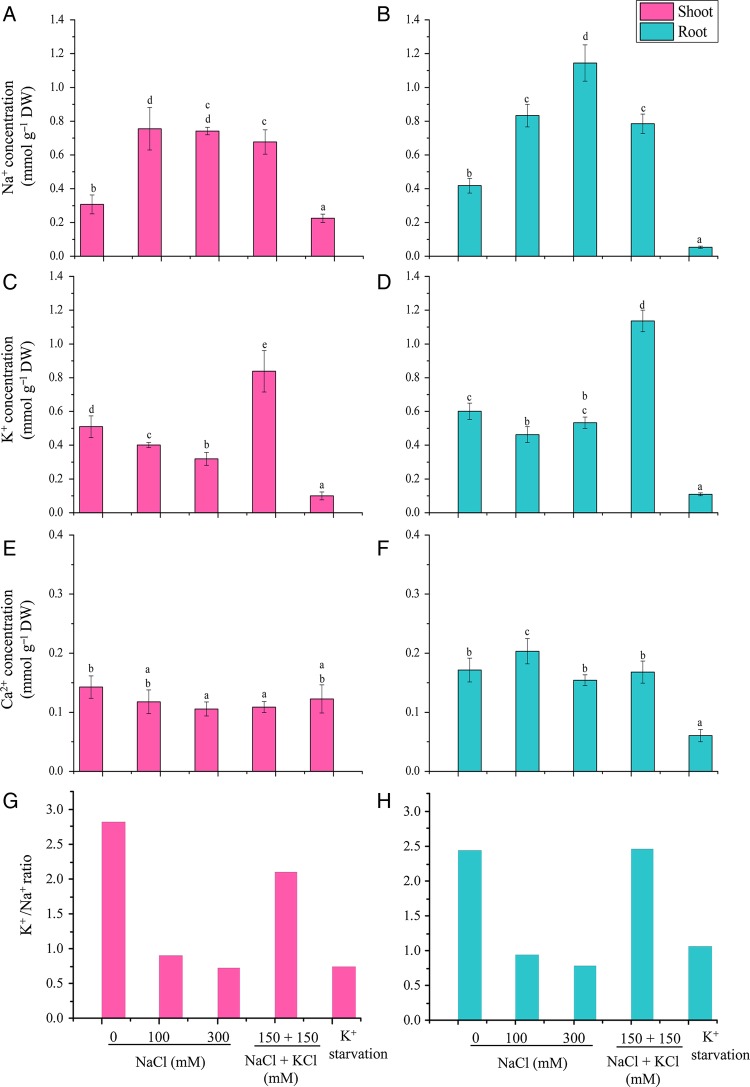
Na^+^, K^+^ and Ca^2+^ contents and the K^+^/Na^+^ ratio in shoot (A, C, E and G) and root (B, D, F and H) tissues of *A. lagopoides* treated with different NaCl concentrations and K^+^ starvation for 6 days. The mean values significantly different at *P* ≤ 0.05 within and among treatments are indicated by different letters. The error bars indicate the SD.

The Na^+^ concentration in shoots increased almost 2.5-fold at 100 mM NaCl when compared with 0 mM (Fig. 4A). The Na^+^ concentration in roots, increased linearly from 0 to 300 mM NaCl ranging from 0.41 to 1.14 mmol g^−1^ DW; however, at 150 mM NaCl + 150 mM KCl the Na^+^ ion concentration remained similar to that in 100 mM NaCl (Fig. 4B).

The K^+^ concentration in the shoots and roots was marginally affected on increasing salinity up to 300 mM NaCl (Fig. 4C and D). High K^+^ accumulation in shoots and roots was observed in plants exposed to 150 mM NaCl + 150 mM KCl treatment. Imposition of K^+^ starvation leads to a significant reduction in cations (Na^+^, K^+^ and Ca^2+^) concentration in shoots and roots of *A. lagopoides*.

The Ca^2+^ concentration in shoots was <0.2 mmol g^−1^ DW and little variation among the treatments. However, in roots the Ca^2+^ was relatively higher (0.20 mmol g^−1^ DW) with 100 mM NaCl treatment. With K^+^ starvation the Ca^2+^ was significantly reduced in roots compared with the other treatments (0.06 mmol g^−1^ DW, Fig. 4F).

The K^+^/Na^+^ regulation is a crucial component for growth and adaptation in plants. At 100 and 300 mM NaCl treatment the ratio was maintained to ∼1.0 (Fig. 4G and H). However, in 150 mM NaCl + 150 mM KCl, the K^+^/Na^+^ ratio was observed to >2 in both shoots and roots.

### Osmoprotectants

**Figure 5.**
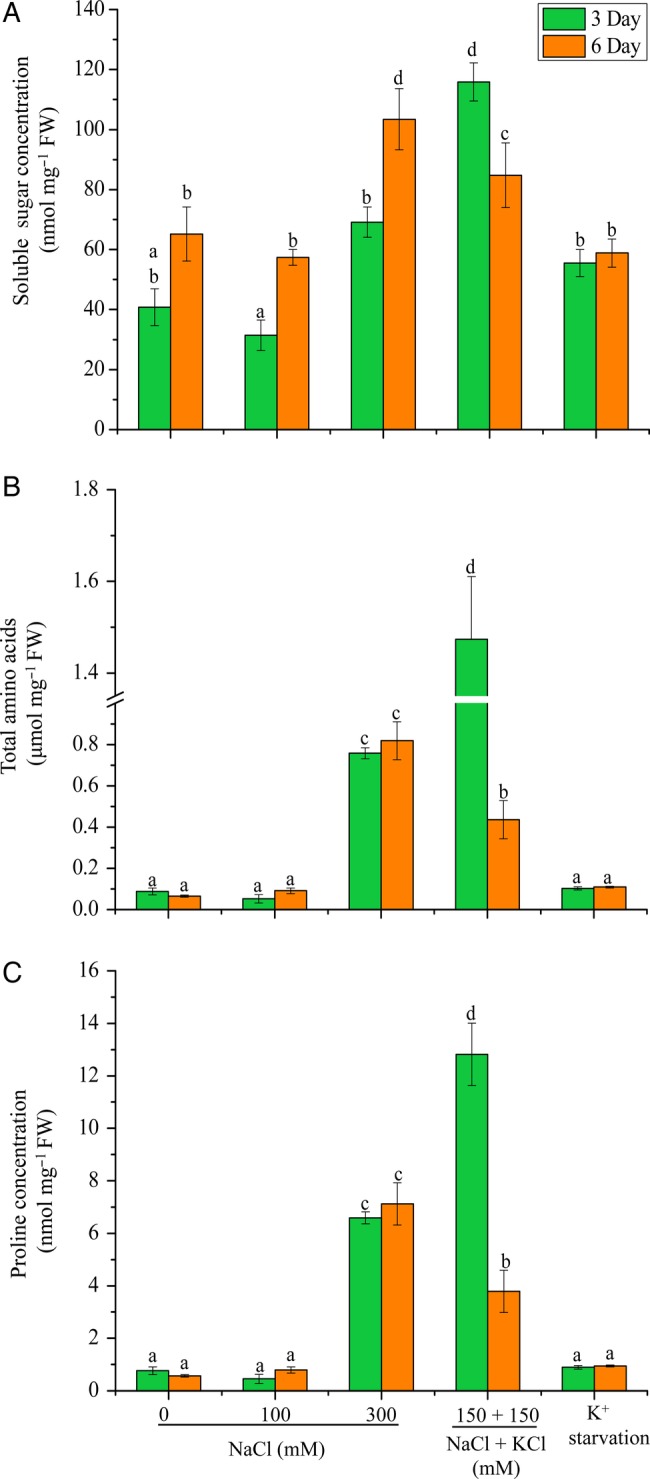
Change in the (A) TSS, (B) TAA and (C) proline contents in shoots of *A. lagopoides* plants treated with 0, 100, 300 mM NaCl, 150 mM NaCl + 150 mM KCl and K^+^ starvation for 3 and 6 days. The mean values significantly different at *P* ≤ 0.05 within and among treatments are indicated by different letters. The error bars indicate the SD.

Similar concentration of TSS, TAA and proline were observed in shoots of 0 and 100 mM NaCl-treated plants (Fig. 5A–C). Increased (compared with 0 and 100 mM treatments) TSS accumulation was observed in 300 mM NaCl at 3 days, which further increased after 6 days (Fig. 5A). High TSS concentration was observed in plants exposed to 150 mM NaCl + 150 mM KCl (3-fold with respect to control plants) after 3 days of treatment; however, it significantly reduced at 6 days. An increase in TAA concentration was visible in plants treated with 300 mM NaCl and 150 mM NaCl + 150 mM KCl at 3 days (Fig. 5B). Proline concentration in 0, 100 mM NaCl and K^+^ starvation was low and showed no significant variation at 3- and 6-day treatments (Fig. 5C). Proline accumulation peaked significantly (8-fold) at 300 mM NaCl with respect to control. Interestingly, the concentration of proline showed 15-fold increase at 150 mM NaCl + 150 mM KCl after 3 days when compared with control plants; however, it decreased after 6 days.

### Oxidative stress marker accumulation

**Figure 6.**
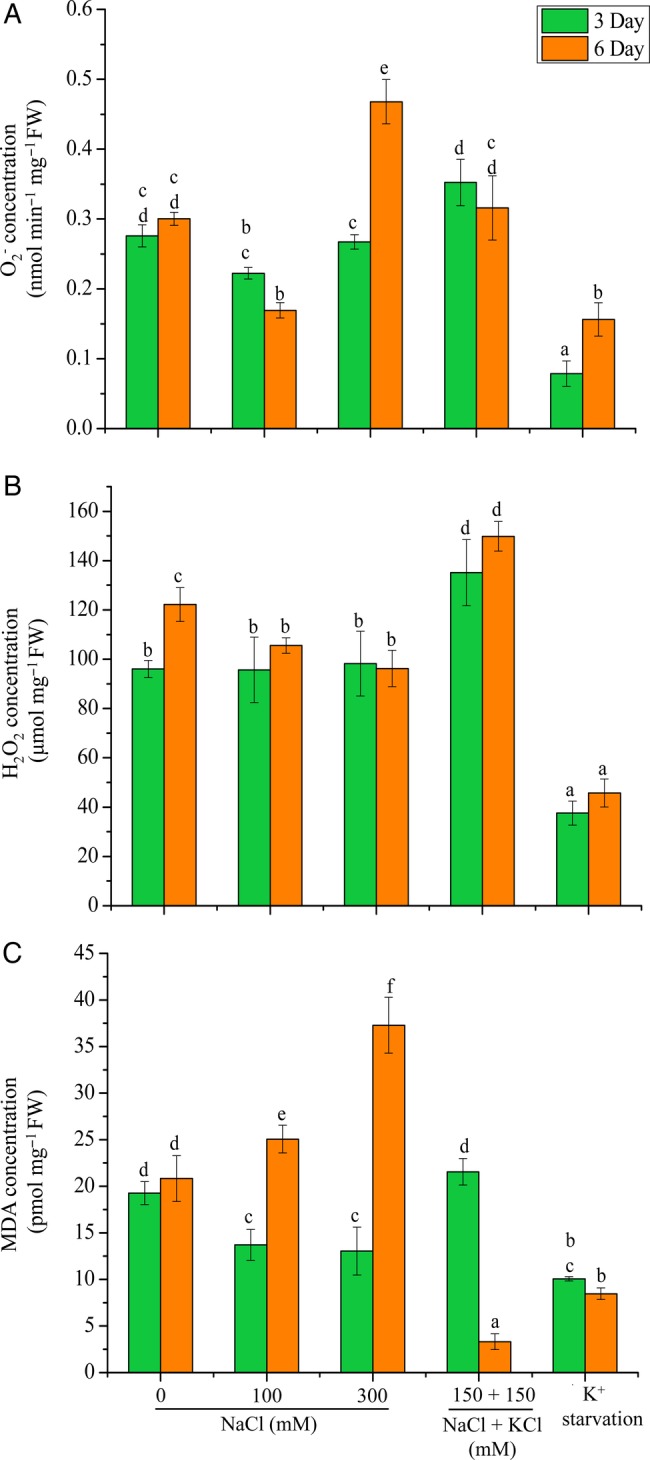
Changes in concentration of oxidative stress markers. (A) Superoxide, (B) peroxide and (C) MDA in shoots of *A. lagopoides* plants treated with 0, 100, 300 mM NaCl, 150 mM NaCl + 150 mM KCl and K^+^ starvation for 3 and 6 days. The mean values significantly different at *P* ≤ 0.05 within and among treatments are indicated by different letters. The error bars indicate the SD.

Superoxide radical accumulation maintained a similar range (0.22–0.352 nmol min^−1^ mg^−1^ FW) at 3-day treatment with 100, 300 mM NaCl and 150 mM NaCl + 150 mM KCl treatments; however, at the 6-day treatment with 300 mM NaCl a significant increase was observed (Fig. 6A). An increase in H_2_O_2_ concentration was observed with 150 mM NaCl + 150 mM KCl treatment for both time durations (Fig. 6B). Malondialdehyde levels increased significantly in plants subjected to 300 mM NaCl for 6 days (Fig. 6C). Contrary to salinity treatments, levels of ${{\text{O}}_{2}}^{-},$ H_2_O_2_ and MDA were significantly low under K^+^ starvation.

### Gene expression analysis of different ion transporters

**Figure 7.**
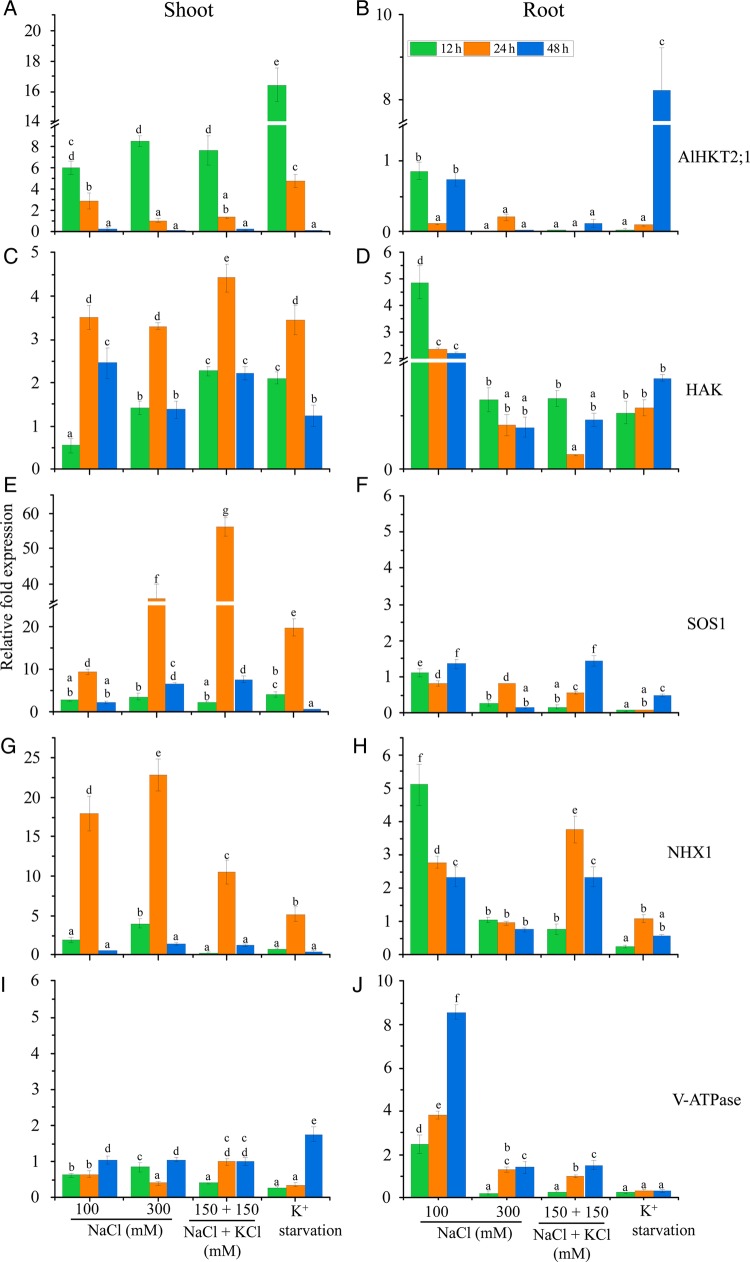
Relative fold expression of different transporter genes from shoot (A, C, E, G and I) and root (B, D, F, H and J) tissues of *A. lagopoides* plants under different stress treatments by real-time PCR. The relative fold expression >1 is considered as up-regulation. The mean values significantly different at *P* ≤ 0.05 within and among treatments are indicated by different letters. The error bars indicate the SD.

The *A. lagopoides* transcripts showed differential regulation of the important transporter genes *AlHKT2;1*, *HAK*, *SOS1*, *NHX1* and V-*ATPase* at 12, 24 and 48 h of stress treatments. In shoot tissue, the *AlHKT2;1* transcript was up-regulated at both 12 and 24 h with all the treatments; however, maximum induction was observed in K^+^ starvation (Fig. 7A). The prolonged treatment (48 h) showed less accumulation of transcripts in all the stress treatments when compared with control (0 mM). In root tissue, *AlHKT2;1* transcript expression was not observed with NaCl stress; however, an 8-fold increase in expression was observed with K^+^ starvation at 48 h (Fig. 7B).

The transcript expression of the *HAK* gene was higher in the shoots at all the treatments compared with control (0 mM) (Fig. 7C). Maximum transcript up-regulation was observed at 24 h in 150 mM NaCl + 150 mM KCl (>4-fold), whereas 3.5-fold higher expression was found in 100 and 300 mM NaCl. The transcript was approximately 3.4-fold higher at 12 h in K^+^ starvation when compared with 0 mM. In roots a maximum increase in transcript accumulation was evident in 100 mM NaCl at 12 h (4.9-fold) (Fig. 7D).

The *SOS1* transcript showed up-regulation with all the treatments in shoot tissue (Fig. 7E). The transcript expression showed early induction at 12 h, which increased further till 24 h, and showed a decrease at 48 h. Interestingly, the 150 mM NaCl + 150 mM KCl showed maximum expression of 56-fold at 24 h. In root tissue, the relative expression of the *SOS1* gene was slightly up-regulated with 100 mM NaCl and 150 mM NaCl + 150 mM KCl (Fig. 7F).

The transcript expression of the *NHX1* gene at 24 h showed maximum accumulation of 18-, 23-, 11- and 5-fold with 100, 300 mM NaCl, 150 mM NaCl + 150 mM KCl and K^+^ starvation, respectively (Fig. 7G). In roots, *NHX1* mRNA accumulation was induced with 100 mM NaCl and 150 mM NaCl + 150 mM KCl (Fig. 7H).

*V-ATPase* expression in shoots was observed only with K^+^ starvation at 48 h (Fig. 7I); however, in roots a transcript up-regulation of 2.5- to 8.5-fold was observed with 100 mM NaCl with varying time durations (Fig. 7J).

## Discussion

Salt stress affects various biochemical and physiological processes like ionic and osmotic homeostasis, pigment production, photosynthesis, carbon partitioning, hormonal regulation, lipid and protein metabolism, consequently whole plant growth and development (Munns 2002). The halophytes have a well-adapted mechanism to thrive and grow in high saline areas which makes them unique compared with glycophytes.

*Aeluropus lagopoides* is one of the important halophytes of family Poaceae and grows commonly in saline marshes. Because of its remarkable adaptability, it has recently gained interest and has been highlighted as an important plant by the Australian new crops website (http://newcrops.com.au/). It displays a remarkable ability to remove excessive salts by excretion through the salt glands present on the leaf surfaces (Naz *et al*. 2009).

Plants maintain water potential gradients between their body and the growth medium by regulating water content and osmotic adjustment (Hasanuzzaman *et al*. 2013). The low water content in addition to accumulation of compatible solutes may be an economical strategy to cope with physiological drought with minimal energy input (Yang *et al*. 2007). Halophytic grasses are known to increase leaf osmolality rather than developing succulence (Rozema and Schat 2013). Glenn (1987) and Marcum and Murdoch (1990) also reported that water content of C4 turf grasses reduced with an increase in salinity. In *A. lagopoides*, the TWC declined in the shoot tissues marginally and maintained a normal range (65–80 %) as reported in other grasses (Tiku and Snaydon 1971). Similarly, water content and succulence of leaves in *Phragmitis communis* (Gorai *et al*. 2007) and *Phragmitis karka* (Abideen *et al*. 2014) were significantly reduced during high saline conditions.

High salt concentration (>40 mM) in the soil evokes Na^+^ toxicity and osmotic stress in plants (Horie *et al*. 2009). Plants counteract the osmotic stress during high salinity by means of osmotic adjustment through turgor maintenance, which is quite low energy consuming but a harmful way to attain osmotic homeostasis (Flowers *et al*. 2015). As the increased accumulation of salt ions in cell cytoplasm leads to the inactivation and degradation of cytoplasmic enzymes (Rozema and Schat 2013). Therefore, plants usually lower the intracellular osmotic potential through biosynthesis of low-molecular, compatible organic compounds like proline, soluble sugars, glycine betaine and polyamines (Rozema and Schat 2013). In the present study, plants subjected to 100 mM NaCl maintained similar concentrations of TSS, TAA and proline as in control plants. Low accumulation of compatible solutes manifests that the plants exposed to 100 mM NaCl do not perceive the stress. No increase in proline content in 150 mM treated plants when compared with control in *A. lagopoides* (Sobhanian *et al*. 2010*b*) also supports the proposed hypothesis. The concentrations of TSS, TAA and proline were increased at higher concentrations of salt at 3 days; however, it decreased at 6 days. The initial increase in the osmolyte content could be due to salt shock, which later declined due to acclimatization. In *Chenopodium quinoa* seedlings, osmotic adjustment during high salt stress was achieved by increase in soluble sugars, proline and glycine betaine (Ruffino *et al*. 2010). Increased accumulation of TSS content under salt stress was observed in the halophytes *Atriplex halimus* (Bajji *et al*. 1998) and *Paspalum vaginatum* (Lee *et al*. 2008). High accumulation of total free amino acids have been reported in shoots of several salt-tolerant plants like sunflower, safflower, *Eruca sativa* and *Lens culinaris* (Fougere *et al*. 1991; Hurkman *et al*. 1991; Ashraf 1994; Ashraf and Fatima 1995; Ashraf and Tufail 1995) in comparison to the salt-sensitive varieties.

Salt stress also evokes oxidative stress leading to increased production of reactive oxygen species (ROS) in the plant cells (Miller *et al*. 2010). High salt concentrations lead to accumulation of toxic Na^+^ and reduced stomatal conductance, which limits the availability of CO_2_ for carbon fixation by the Calvin cycle (Ozgur *et al*. 2013). Therefore, incident radiation for photosynthesis is not completely utilized by the available intracellular CO_2_ and leads to the formation of ROS (Miller *et al*. 2010). In present study, the superoxide concentration was similar at 100 (3 and 6 days) and 300 mM NaCl (3 days) treatment. However, it increased at longer period, this may be because plants do not perceive stress at an early time point. The ion content analysis also showed no change in different stress time. Hydrogen peroxide was also found almost similar at 100 and 300 mM NaCl when compared with control.

Halophytes have evolved a unique ability to protect cells against oxidative stress. *Atriplex lentiformis*, *A. lagopoides* change its mode of carbon fixation from the C3 to C4 pathway in response to salinity stress (Meinzer and Zhu 1999; Sobhanian *et al*. 2010*a*). The shift in the carbon fixation pathway from C3 to C4 during salt stress leads to reduced production of ROS (Hurst *et al*. 2004). Moreover, further studies related to the photosynthetic pathways during moderate (100 mM NaCl) and high (300 mM NaCl) salt stress can provide more insight on a plant's physiology during salt stress.

The high MDA content observed with 300 mM NaCl at 6 days reduced significantly by combined stress of NaCl and KCl, indicating the involvement of K^+^ in reducing the stress. Sobhanian *et al*. (2010*b*) have reported progressive increase in superoxide dismutase activity and no change in catalase and ascorbate peroxidase levels in *A. lagopoides* during high salt stress. Besides osmotic regulation, organic osmolyte plays a crucial role in the protection of cellular enzymes and plasma membrane stability and also acts as ROS scavengers. In this context, the increase in the proline concentration in plants subjected to 300 mM NaCl and 150 mM NaCl + 150 mM KCl may have enabled the plants to reduce ROS accumulation and minimize lipid peroxidation.

Halophytic grasses maintain a high K^+^/Na^+^ ratio in the cytosol and also exhibit comparatively higher K^+^ over Na^+^ selectivity when compared with dicot halophytes (Flowers and Colmer 2008; Munns and Tester 2008). During salt stress, they may employ more efficient mechanisms involved in K^+^ homeostasis and Na^+^ exclusion than relying on ion sequestration mechanisms generally used by dicot halophytes (Rozema and Schat 2013). The increased Na^+^ concentration in shoots at 100 mM NaCl remained steady even at 300 mM NaCl and 150 mM NaCl + 150 mM KCl (Fig. 4). This constant Na^+^ concentration may be maintained by a higher rate of Na*^+^*secretion, which is evident by the increased number of the crystals (Fig. 1D). The high Na^+^ content in roots concomitantly increased by increasing the salt concentration may be because of weak control of Na^+^ ions influx (Sobhanian *et al*. 2010*b*). Plants regulate the efflux of Na^+^ from the cytoplasm mainly by the activity of vacuolar (NHX1; Apse and Blumwald 2007) and plasma membrane Na^+^/H^+^ antiporters (SOS1; Shi *et al*. 2002). The *SOS1* gene has been isolated from several plants like *Arabidopsis*, rice, wheat, tomato, *Thellungiella salsuginea* etc. (Yamaguchi *et al*. 2013). Overexpression and knockout studies of the *SOS1* gene highlight the involvement of this gene in salt tolerance. The disruption of the *SOS1* gene activity renders the plant more sensitive to salt stress and overexpression of *AtSOS1*, *T. salsuginea SOS1* and *OsSOS1* conferred salt tolerance in *Arabidopsis* plants (Yamaguchi *et al*. 2013). In the present study, the high transcript accumulation of *SOS1* gene was observed in shoots to maintain low Na^+^ by exporting it in the apoplast. Shi *et al*. (2002) observed that *Arabidopsis sos1* mutant plants accumulated more Na^+^ in the shoot and xylem sap than wild type, thereby suggesting its role in Na^+^ retrieving from the xylem stream in response to severe salt stress. The *SOS1* gene from non-salt-secreting halophyte *Thellungiella halophila* (*ThSOS1*) showed an increased transcript in both the roots and shoots of plants exposed to salt stress (Vera-Estrella *et al*. 2005). Ding *et al*. (2010) have also proposed that *SOS1* may play an important role in salt secretion process by transporting Na^+^ from cytoplasm of secretory cells to the exterior. The *NHX1* have been identified from different halophytes; *Suaeda salsa*, *Atriplex gmelini*, *Mesembryanthemum crystallinum*, etc. (Flowers and Colmer 2008). Apse *et al*. (2003) reported that the *Arabidopsis atnhx1* mutant showed significantly reduced vacuolar Na^+^/H^+^ antiport activity and displayed Na^+^ sensitivity, highlighting the function of vacuolar NHXs in Na^+^ accumulation under salinity stress. In this study, the *NHX1* gene showed up-regulation in both shoots and root tissue under a salt stress condition. The combined stress (150 mM NaCl + 150 mM KCl) enhanced *NHX1* transcripts in both in shoots and roots, highlighting its role in K^+^ uptake also.

Potassium is the vital macronutrient for plants and plays indispensable roles in a number of physiological processes (Anschütz *et al*. 2014). Halophytes exhibit high K^+^ over Na^+^ selectivity in order to maintain a high K^+^/Na^+^ ratio in the cytosol. Two gene families encoding the transmembrane K^+^ uptake systems have been recognized in plants, the Shaker K^+^ channel family (Very and Sentenac 2003) and the HAK/KUP/KT K^+^ transporter family (Gierth and Mäser 2007). In monocots a third family namely HKT has been identified, which is also known to mediate K^+^ transport (Corratgé-Faillie *et al*. 2010).

*Aeluropus lagopoides* maintained the K^+^/Na^+^ ratio in shoots and roots during 100 and 300 mM NaCl stress, probably due to the strong activity of K^+^ transporters like *HKT* (Class II) and *HAK* in this halophyte. Our results are in line with Gulzar *et al*. (2003), where they reported that *A. lagopoides* plants retained constant K^+^ content with moderate salt stress. Other halophytic grasses like *Phragmites karka* (Abideen *et al*. 2014) and *Puccinellia tenuiflora* (Yu *et al*. 2011) showed elevated cytoplasmic K^+^/Na^+^ ratios under high salinity.

In this study, the *AlHKT2;1* transcript was expressed in shoots in response to NaCl treatment and K^+^ deficiency. However, in roots delayed expression was observed only with K^+^ starvation, indicating its late involvement in roots, at a time when its expression in shoots is down-regulated. The functional characterization of *AlHKT2;1* using the K^+^ uptake yeast mutant showed that it mediates K^+^ uptake and alleviates salt stress in yeast (Sanadhya *et al*. 2015). Similarly, the *PutHKT2;1* gene showed enhanced transcript accumulation during K^+^ starvation or high NaCl stress (Ardie *et al*. 2009). The *TsHKT1;2* from *T. salsuginea* also showed an enhanced transcript regulation in high NaCl conditions (Ali *et al*. 2012). In the present study, the higher expression of the *HAK* transcript during salt stress and K^+^ starvation can be correlated to its role in salt tolerance. Su *et al*. (2002) also observed that the transcripts of the *HAK* gene in *M. crystallinum* were up-regulated in salt stress and K^+^ starvation. Also, *HAK* from *A. littoralis* enhanced the Na^+^ tolerance of the mutant yeast cells (Su *et al*. 2007). *OsHAK1* transcript accumulation was more influenced by Na^+^ concentration than that of K^+^ (Wu *et al*. 2009). These studies emphasize that *AlHKT2;1*, HAK transporters mediate K^+^ transport to maintain a suitable Na^+^/K^+^ ratio in cytosol during NaCl stress for different physiological processes.

Short-term K^+^ starvation (6 days) did not affect TWC, TSS, TAA and proline; however, the Na^+^, K^+^ and Ca^2+^ contents were significantly reduced in comparison with control in our study. Similarly, Wu *et al*. (2009) reported that in rice the elimination of K^+^ supply for 9 days did not significantly affect the growth of roots or shoots. Although K^+^ is necessary for the plant growth and physiology; however, it appears that the plant can sustain the short duration of K^+^ starvation. Future studies will be undertaken to study the long-term effect of K^+^ starvation on biochemical parameters and transcript regulation of different ion transporters.

## Conclusion

*Aeluropus lagopoides* was able to survive (for 6 days) under moderate and high salinity by accumulating high Na^+^ in the roots and secretion of excess ions from leaf sheath as well as the adaxial and abaxial leaf surfaces through salt glands. At high salt concentration, the level of different organic and inorganic osmolytes gets elevated to prevent toxicity and maintained a sustainable K^+^/Na^+^ ratio. In high salt concentration, these plants showed accumulation of superoxide radicals and MDA. The transcript profiling of transporter genes revealed the up-regulation of K^+^ transporters like *AlHKT2;1* and *HAK* in coordination with the Na^+^ transporters for Na^+^ compartmentalization towards Na^+^ and K^+^ homeostasis and probable salt secretion. Short-term K^+^ starvation did not significantly affect the physiological mechanisms; however, the K^+^ transporters *AlHKT2;1* and *HAK* were up-regulated. Therefore, plausibly it can be mentioned that different transporters work together in this plant and maintain a balanced ion concentration to survive high salt concentration.

## Sources of Funding

The study was supported by funding from Council of Scientific and Industrial Research (CSIR), New Delhi, India.

## Contributions by the Authors

P.S. carried out all the experiments, P.A. involved in gene expression analysis and P.K.A. coordinated the experiments. All authors approved the final manuscript.

## Conflict of Interest Statement

None declared.
